# The renaissance of life near the boiling point – at last, genetics and metabolic engineering

**DOI:** 10.1111/1751-7915.12463

**Published:** 2016-12-08

**Authors:** Michael W.W. Adams, Robert M. Kelly

**Affiliations:** ^1^ Department of Biochemistry and Molecular BiologyUniversity of GeorgiaAthensGA30602‐7229USA; ^2^ Department of Chemical and Biomolecular EngineeringNorth Carolina State UniversityRaleighNC27695‐7905USA

## Abstract

We discuss here the prospects for biotechnology of extreme thermophilic microorganisms.

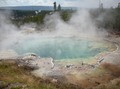

Imagine microbial biology and biotechnology without genome sequences and genetic tools. On top of that, consider working with microorganisms that do not grow at ambient temperatures or on solid media. Yet another challenge – these microorganisms inhabit extreme thermal environments that can be dangerous and expensive to access (Fig. 1). These were the challenges facing pioneering microbiologists, such as Thomas Brock, Holger Jannasch, Wolfram Zillig and Karl Stetter, in the latter half of the 20th century as they established extreme thermophily in the lexicon of modern microbiology. By the end of the century, numerous microbes had been described which could grow near, and even above, the boiling point of water (see Fig. 2). They were isolated from continental hot springs and shallow marine vents, and many were discovered in deep sea hydrothermal environments several kilometres below sea level (Stetter *et al*., 1990). Moreover, most of these microbes were classified as archaea, with only a very few bacterial species known able to grow optimally at temperatures above 80°C. These organisms encompass a range of metabolic modes. They include those capable of aerobic and anaerobic respiration; autotrophic and heterotrophic metabolism; sugar and peptide fermentation; reduction in elemental sulfur, sulfate, thiosulfate, ferric iron, arsenate and nitrate; oxidation of metals and metal oxides; and the conversion of hydrogen gas and carbon dioxide to methane.

**Figure 1:**
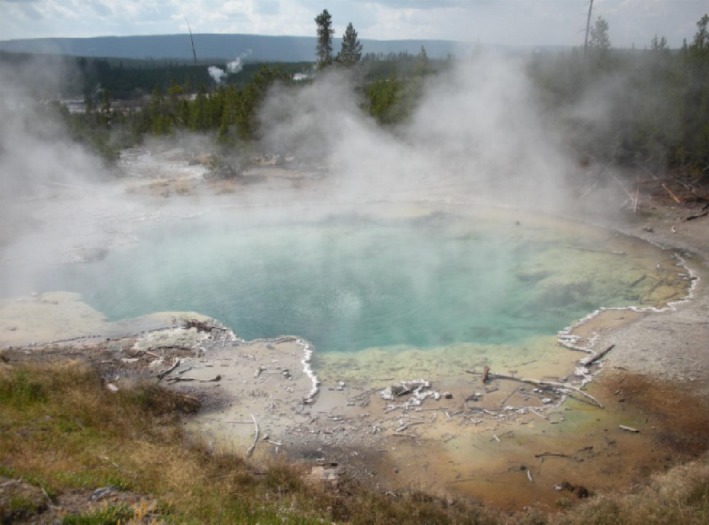
Hot spring in Yellowstone National Park, USA.

**Figure 2:**
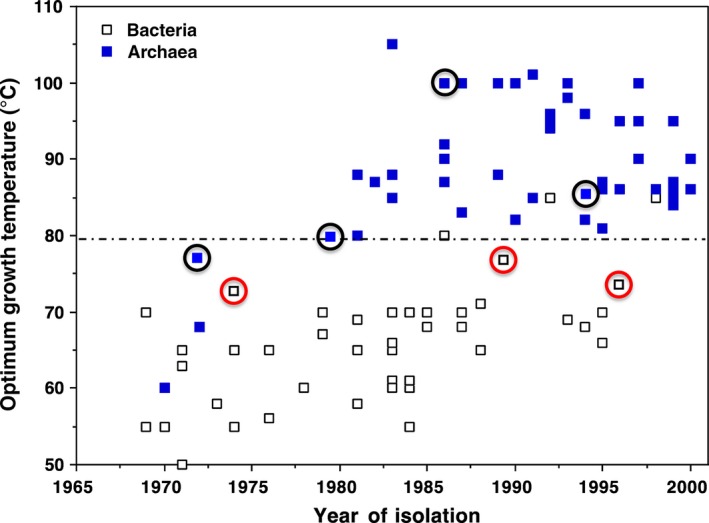
Isolation of thermophilic species in the 20th century. Those that now have genetic systems are circled (red for bacteria and black for archaea).

In the mid‐1990s, the enormous interest in these extremely thermophilic microbes, and also in the archaeal domain of life, is well illustrated by the fact that three of the first dozen genomes to be sequenced were from thermophilic archaea (*Methanococus, Archaeoglobus* and *Methanobacterium*), with those of the most thermophilic bacteria determined shortly thereafter (*Thermotoga* and *Aquifex*). Their genomes were typically smaller (~2 Mb) than those of the model mesophilic bacteria (3–4 Mb), and their sequences provided insights into how these microbes from odd places fit into the evolutionary scheme of life. They also enabled the expression of numerous genes from these organisms in microbial models, such as *Escherichia coli*, opening up the window on studies of protein stability and enzyme catalysis at temperatures even above 100°C. Genome sequences enabled global transcriptional studies that, coupled with biochemical analyses, provided many novel physiological and metabolic details of extreme thermophily, although general rules of life at extreme temperature remained elusive and still do to this day. Stabilizing a protein above the boiling point involves rather minor changes relative to its mesophilic counterpart, and the mechanisms involved are generally unique to a particular protein. Moreover, even with genome sequence information in hand, biotechnological opportunities at extreme temperatures were restricted to single‐step biocatalysis using recombinant enzymes.

Realizing the true biotechnological potential of microbes that thrive near above the boiling point of water has been greatly hampered by the inability to genetically manipulate them, but this has recently changed. Specifically, genetic systems reproducible in more than one laboratory have now been developed for two genera that grow optimally above 80°C, the anaerobic archaea *Thermococcus* and *Pyrococcus* (see Fig. 2), both of which grow by fermenting sugars and peptides. Techniques to grow these organisms on plates at high temperature in the absence of oxygen were developed with *T. kodakorenesis* (T_opt_ ~85°C) over a decade ago through the pioneering work of Imanaka and Atomi (Sato *et al*., 2003). More recently, these methods have been applied to other *Thermococcus* species (Kim *et al*., 2010; Lim *et al*., 2014) and to *P. furiosus* (T_opt_ ~100°C)*,* the first for a microbe to grow at or above the boiling point of water (Bridger *et al*., 2012).

Genetic studies to date with *Thermococcus* and *Pyrococcus* have mainly focused on providing insights into their physiology and metabolism and the homologous overproduction of affinity‐tagged enzymes (McTernan *et al*., 2014). However, recent reports demonstrate metabolic engineering of these organisms for biotechnological purposes. For example, heterologous gene expression in *Pyrococcus* has enabled it to use carbon monoxide as an energy source (Schut *et al*., 2016) and to generate from sugars the biofuel n‐butanol (Keller *et al*., 2015) and the industrial building block 3‐hydroxypropionate (3HP) (Keller *et al*., 2013). Through bioengineering approaches, 3HP production near the gram per litre scale is now possible (Hawkins *et al*., 2015; Lian *et al*., 2016). Kinetic modelling promises to further increase bioproduct formation (Loder *et al*., 2016). In addition, the first example of the industrial application of a genetically engineered extreme thermophile, *T. onnurineus* (T_opt_ ~85°C)*,* was recently reported: a recombinant strain of this organism was optimized for the conversion of carbon monoxide produced from processing steel into hydrogen gas at very high efficiency (Simon *et al*., 2015). These studies demonstrate what can be achieved when prior extensive studies on the metabolism and enzymology of life near 100°C are utilized for biotechnological purposes and the production of useful products.

Bioprocessing at temperature near and above 80°C can have important advantages over near‐ambient operations. Highly genetically modified microorganisms usually have a fitness disadvantage and can be easily overtaken in culture when contaminating microbes are present. The high growth temperature of extreme thermophiles precludes growth or survival of virtually any contaminating organism. This reduces operating costs associated with reactor sterilization and maintaining a sterile facility. In addition, at industrial scales, heat production from microbial metabolic activity vastly outweighs heat loss through bioreactor walls such that cooling is required. Extreme thermophiles have the advantage that non‐refrigerated cooling water can be used if needed, and heating requirements can be met with low‐grade steam typically in excess capacity on plant sites. In fact, in the process in which the metabolically engineered *T. onnurineus,* which produces at 85°C hydrogen gas from the CO generated in steel mills, has no sterilization requirements, no cooling needs, and uses waste heat from the plant site and uses sea water to make up water losses (*T*. *onnurineus* is of marine origin). In fact, in a process based on metabolically engineered *T. onnurineus*, production of hydrogen gas at 85°C from CO generated in steel mills has no sterilization requirements, no cooling needs, uses waste heat from the plant site, and uses sea water to make up water losses (*T. onnurineus* of a marine organism).

Genetic systems are also available for four genera of microbes that grow optimally in the 70 – 80°C range (see Fig. 2), and these offer a wider spectrum of metabolic diversity than *Thermococcus* and *Pyrococcus* species. All are heterotrophs and include the aerobe *Thermus,* the acidophilic aerobe *Sulfolobus,* and the anaerobes *Thermoanerobacterium* and *Caldicellulosiruptor*. Of these, *Caldicellulosiruptor* species probably have the most potential in the biotechnological field because of their ability to break down and grow on the carbohydrate polymers cellulose and hemicellulose (xylan), the primary components of plant biomass. Indeed, these organisms can degrade wood and grasses without any thermochemical pre‐treatment (Kataeva *et al*., 2009; Zurawski *et al*., 2015). Recent advances in the genetic system for these organisms (Lipscomb *et al*., 2016) bode well for their future utilization in biomass to biofuel conversion processes.

So, a renaissance of sorts is at hand in the field of extreme thermophily that offers engineering these organisms for biotechnological processes. One can anticipate that this field will soon take advantage of the latest developments in genetic manipulations, such as Tnseq and CRISPR, as well as the full spectrum of tools in systems biology. The future looks bright for these remarkable organisms that thrive at the upper temperature limits of life.
